# Effect of herbal mouthrinsein dental ultrasonic scalers among Indians

**DOI:** 10.6026/973206300191104

**Published:** 2023-11-30

**Authors:** Ankita Agrawal, Shilpa Keerthipati, Sudhalatha Sreerama, Deepika Singla, Sonu Acharya, DhavalNiranjan Mehta, Santosh Kumar, Kapil Paiwal

**Affiliations:** 1 Department of Conservative and Endodontics, Buddha Institute of Dental Sciences and Hospital, Patna, Bihar, India; 2 Department of Orthodontics, Gitam Dental College and Hospital, Visakhapatnam, India; 3 Gitam Dental College and Hospital, Visakhapatnam, India; 4 Department of Conservative Dentistry & Endodontics, Desh Bhagat Dental College & Hospital, Mandi Gobindgarh, Punjab, India; 5 Department of Pediatric and Preventive Dentistry, Institute of Dental Sciences, Siksha Anusandhan (Deemed to be) University, Bhubaneswar, India; 6 Department of Oral Medicine and Radiology, Narsinbhai Patel Dental College and Hospital, Sankalchand PatelUniversity, Visnagar, Gujarat, India; 7 Department of Periodontology, Karnavati School of Dentistry, Karnavati University, Gandhinagar, Gujarat, India; 8 Department of Oral & Maxillofacial Pathology, Daswani Dental College & Research Center, Kota, India

**Keywords:** Chlorhexidine, povidone iodine, cinnamon extract, curcumin, ultrasonic coolant, reduction, bacterial load and dental aerosols

## Abstract

The use of herbal mouthrinse is gaining momentum in recent years. Therefore, it is of interest to evaluate the effect of 2 herbal mouthrinse (curcumin, cinnamon) in comparison with2 conventional mouthrinse (povidone iodine, chlorhexidine) when used as coolant in dental ultrasonic scalers. Hence, 200 participants were included in this study. Analysis of gingival index, periodontal index at baseline and one month follow up was completed. The inhibitory effects of both conventional and herbal mouth rinse in gingival health are similar. However, cinnamon and curcumin owing to its minimal adverse effects and low cost is useful as an alternative to chlorhexidine for reducing bacterial load in dental aerosols produced due to ultrasonic scalers.

## Background:

Aerosols produced when using an ultrasonic scaler or the air rotor of a dental chair contains droplet nuclei particles. These aerosols remain in the environment for extended periods of time [1,2]. These aerosols pose a risk to both patients and dental professionals for the development of contagious diseases [3, 4]. Numerous studies conducted over the past 15 years have shown that the amount of germs aerosolized during clinical practise can be reduced when antimicrobial solutions are utilised as pre-procedural rinses [5, 6]. Numerous studies on the application of preprocedural antibacterial mouthwashes have produced promising findings [7,8]. Water is used as a coolant during the usual practise of ultrasonic debridement and performs a number of other functions. It helps to shorten the process by lowering frictional heating, cleaning the treated region to improve visibility, and quickening the process [9,10]. However, using freshwater as an ultrasonic cooler during plaque removal treatments raises the danger of contamination, the formation of aerosols, and the possibility of transitory bacteremia [11,12]. So, as a coolant, several chemotherapeutic drugs have been used to prevent this. Pre-procedural rinsing with 0.12% chlorhexidine (CHX) gluconate lowers the amount of facultative and aerobic oral flora [13, 14]. When utilised as a pre-procedural rinse, povidone iodine (PVP) keeps the gingival surface microorganisms from growing during the preventive process. Dental professionals are more likely to contract infectious infections, particularly those affecting the respiratory system [15,16]. The most commonly utilised device in a dental setup is the ultrasonic scaler, which is the main means of dental aerosol output. When administered as a preprocedural rinse, chlorhexidine gluconate decreases the amount of facultative and aerobic oral flora [17]. Periodontal pathogens like *Porphyromonas gingivalis*, *Aggregatibacteractino mycetemcomitans* and others may be killed by diluted PVP in vitro in a matter of fifteen seconds of contact. While bacteria and yeast get killed 5 minutes of contact in *in vivo* condition [18].

Despite other antimicrobials having been conventionally evaluated and used as mouthwashes, chlorhexidine has been the industry standard [17,18]. However, using it can have some negative side effects, including discoloration of teeth and dental work, dryness of the mouth, oral discomfort, enhanced supragingival calculus development, and altered taste [19,20]. Chlorhexidine mouthrinses are also believed to affect the strength of bond of polycarbonate orthodontic brackets with enamel. Due to their safety, the use of herbal compounds as medicines has recently attracted significant attention in both medicine and dentistry across the globe. Medical study is increasingly placing more emphasis on turmeric, one of the most used home treatments. Curcumin, a hydrophobic polyphenol derived from the root system of curcumin longa, is the primary component of turmeric [11]. In addition to promoting healing of wounds, curcumin has immunomodulatory, antioxidant, antibacterial and anti-inflammatory effects [12]. Curcumin modulates NF- -κβ in diseases of periodontium. Numerous cytokines are suppressed by curcumin, and several enzymes, including stimulated nitric oxide synthase and lipoxygenase, are downregulated. They have an extra benefit in treating periodontal disease since they are strong hunters of oxygen species that are reactive. Numerous periodontopathogens are inhibited by curcumin in a way that depends on the dose [13]. 

There have been very few research investigations assessing the effects of herbal mouthrinses. One such substance is cinnamon extract. Dried bark of *Cinnamomum zeylanicum* and *Cinnamomum aromaticum* [21] is often referred to as cinnamon and is used to make many kinds of spicy candies, chocolate and alcoholic beverages [22]. Additionally, cinnamon has been utilised in traditional Chinese medicine, which dates back about 4,000 years, as a neuroprotective substance and to treat diabetes [23, 24]. Additionally, inflammation, gastrointestinal issues, and urinary infections have all been treated using cinnamon as a health-promoting agent [25,26] Because of its antimicrobial qualities, particularly its antibacterial action, cinnamon may also be used medically. This data supports the use of extract of cinnamon as a mouthwash in the management of gingivitis and for promoting gingival health [27,28]. Therefore, it is of interest to evaluate the effect of 2 herbal mouthrinse (curcumin, cinnamon) in comparison with 2 conventional mouthrinse (povidone iodine, chlorhexidine) when used as coolant in dental ultrasonic scalers among Indians.

## Methods and Materials:

A three-category parallel layout, placebo-controlled research investigation carried out at single centre was the format of this research. Participants in the research project were chosen from the periodontology outpatient division, and it lasted for six months.

## Sample size:

The sample size in each category was 40. Total participants were 200.

## Selection standards:

[1] The following were the study's inclusion guidelines

[2] Study patients with a healthy framework

[3] Participants made a single-sitting complete mouth scaling request

[4] Study participants must have at least twenty permanent teeth

[5] Individuals with a gingival index (GI) value of 2-3 and moderate-to-severe gum inflammation

## The following were the study's exclusion standards:

[1] Past three months of oral prophylaxis

[2] Women who are pregnant or nursing

[3] Smokers

[4] Existence of any systemic illness

[5] Study participants who have used either antibiotics or NSAIDs within the previous 9-11 weeks

## Randomization and patient selection:

One evaluator who was unaware of study design carried out the entire clinical examination and evaluation process. Oral mucosa evaluation and gingival health inspection have been incorporated in the clinical evaluation. In the first session, the investigation's participants were initially examined to obtain their GIas well as plaque index (PI) scores. For the purpose of this research, 200 people with moderate-to-severe gingivitis of both sexes, ages 18 to 55, which were willing to take part in the investigation and who also had GI and PI scores of 2-3, were chosen. Following a follow-up of one month, participants were brought back in for a single clinical parameter assessment. A computer-generated random sequence table was used by one examiner to assign the patients at random to one of the three groups while another examiner administered the treatment. The study includes the following five categories:

[1] Category I: Forty participants using chlorhexidine as a coolant in ultrasonic scaling (n=40)

[2] Category II: Forty participants using the extract of cinnamon as a coolant in ultrasonic scaling (n=40)

[3] Category III: Forty participants using povidone iodine as a coolant in ultrasonic scaling (n=40)

[4] Category IV: Forty participants using curcumin as a coolant in ultrasonic scaling (n=40)

[5] Category V: Forty participants using distilled water (DW) as cooling agent in ultrasonic scalers (n=40) (Control)

## Experimental solutions

CHX digluconate 0.2 percent, povidone iodine 1%, powdered curcumin (Sigma Aldrich, USA), cinnamon extract were employed as the subject of the research's material of testing. The concentration of povidone iodine used was 1%. The concentrations of CHX applied in this research were 0.2%. The concentrations of CUR evaluated in this research were 0.12%.

Control: Distilled water was taken as control

## Clinical approach:

All treatment operations were carried out in a closed operating room with a fumigation facility. Ethyl alcohol (70%) was used to sanitise the operator's equipment before the operation was performed. The ultrasonic device was turned on and drained for two minutes before to the treatment to remove polluted water that had accumulated nightly in the waterlines. A blood plate made from agar was placed on the surface of one region for a period of fifteen minutes before surgery. This was then put through a microbiological analysis to see if there were any contaminants from the environment in the dental office. Only until the operator was certain that there was no contamination from the environment visible on the agar plate, did the process on the patients start. For the investigation, dental chairs featuring self-contained water systems were used. The waterlines of dental units (DUWL) were amended with the aforementioned experimental chemicals. All of the patients received single-sitting, 20-minute ultrasonic scaling utilising an Ultrasonic Scaler. Each scaling process involved the application of a saliva remover. The participants in each category were questioned about any pain they experienced, such as a change in taste or sensation of burning following the debridement operation, once the surgery was over. Study participants were requested to notify the dental clinic if any side effects developed following treatment.

## Position of agar plates:

Based on the results of an earlier research study for data repeatability, the placement of the plates made from agar was decided. For every single treatment category, the three separate graphical positions of the blood plates made of agar were chosen in the operating room, and established distances between the agar plates and the reference point-the patient's mouth-were also maintained. Both right and left side were each provided with two separate plates for oxygenated culture, accordingly. To see if the number of colonies formed was almost comparable, two plates were purposefully put.

## Microbial examination:

## Aerosol analysis:

In each of the five categories, the aerosols produced by the ultrasonic device were gathered on 2 plates of blood agar that were positioned at three distinct locations, every plate within a distance of one foot. Plates from every location were subjected to incubation aerobically for a period of 48 hours after the specimens were collected.

## Examination of the biofilm on dental office waterlines

For each of 5 categories, a peeling was used to get a small amount of biofilm from a DUWLs tubing. The sample spent 24 hours in saline. Following that, utilising a cotton bud, 0.1 ml of the aforementioned saline was deposited on the surface of agar based plates for each category of participants. The plates were subsequently left to incubate under aerobic conditions for period of 48 hours. The total amount of colony-forming units (CFUs) that were visible on agar plates was used to indicate the total amount of bacterial colonies that were identified using the traditional bacterial enumeration methodology.

## Statistic evaluation

SPSS version 20 software (USA) was used to statistically analyse the findings for CFUs. After ensuring that the data were distributed normally, the ANOVA test was applied to variables that were continuous. The data's distribution of Gaussian variables was verified using the Bartlett method. ANOVA was also used for the intergroup assessment of the clinical outcomes (GI and PI), whereas Student's t-test was used for the intragroup investigation. The threshold for statistical significance was set at P < 0.05.

## Results:

The mean value of gingival index in category I at baseline and one month follow up was 2.31±0.21 and 0.27 ±0.15 respectively. The mean value of gingival index in category II at baseline and one month follow up was 2.23±0.18 and 0.20±0.04 respectively. The mean value of gingival index in category III at baseline and one month follow up was 2.29±0.21 and 0.27 ±0.15 respectively. The mean value of gingival index in category IV at baseline and one month follow up was 2.24±0.18 and 0.21 ±0.04 respectively. The mean value of gingival index in category V at baseline and one month follow up was 2.32±0.18 and 0.58±0.19 respectively (Table 1).

The mean value of periodontal index in category I at baseline and one month follow up was 2.42 ± 0.23 and 1.10 ±0.10 respectively. The mean value of periodontal index in category II at baseline and one month follow up was 2.38±0.31 1.06±0.12 respectively. The mean value of periodontal index in category III at baseline and one month follow up was 2.42 ±0.21 and 1.12 ±0.10 respectively. The mean value of periodontal index in category IV at baseline and one month follow up was 2.38±0.34 and 1.07±0.12 respectively. The mean value of periodontal index in category V at baseline and one month follow up was 2.51±0.07and 1.31±0.14 respectively (Table 2).

In each category there was decrease in values of gingival index and periodontal index at one month follow up during intra group comparison. The difference in values of gingival index and periodontal index at baseline and one month follow up was meaningful statistically in all categories (p <0.05).

When there was inter group comparison among five categories then the values were maximum in category V (distilled water) and minimum in herbal mouthrinse (cinnamon and curcumin). The values of conventional mouthrinse (chlorhexidine and povidone iodine) was lesser than category V (distilled water) but greater than herbal mouthrinse (cinnamon and curcumin) (category V> category I= category III> category II =category IV). There was improvement in gingival health in both conventional and herbal mouthrinse study participants. However, the improvement was greater in chlorhexidine and povidone iodine. There was no difference statistically significant difference.

It was observed that CFU values were maximum in control (category V) (distill water group) at all locations as compared to category I, II, III, IV. The findings were significant statistically (p <0.05). The CFU values were low in category II (cinnamon) and category IV (curcumin) at chest area. The CFU values were low in category I (chlorhexidine) and category III (povidone iodine) at left and right side of chest. The findings were meaningful statistically (p<0.05). The maximum number of CFU was observed at chest area of patient in all three experimental materials. The findings were meaningful statistically (p<0.05).

The value of CFU in waterline of dental units in category I was 361.81 ± 15.21. The value of CFU in waterline of dental units in category II was 279.81 ±27.81. The value of CFU in waterline of dental units in category III was 354.81 ± 16.32. The value of CFU in waterline of dental units in category IV was 281.92 ±28.92. The value of CFU in waterline of dental units in category V was680.1 ±42.51.The maximum CFU was in distilled water category while it was minimum in cinnamon and curcumin category. The findings were meaningful statistically. The values were greater in chlorhexidine category and povidone iodine category as compared to cinnamon and curcumin (Table 3). However, the difference in values in cinnamon, curcumin as compared to chlorhexidine and povidone iodine did not show statistically significant difference.

## Discussion:

Several dental procedures result in the production of aerosols. However, there is an increased risk of infection transfer due to aerosols created during ultrasonic cleaning procedures since blood and live bacteria are present subgingivally [12, 13]. In a dental office, a variety of techniques are employed to lessen infection caused by microorganisms. This includes using pre-procedural mouthwashes, using personal barriers of protection, immunizing dental personnel, and disinfecting surfaces [14, 15]. Chlorhexidine has been the industrial standard even though other antimicrobials have been conventionally examined and used as mouthwashes. However, it may have certain unfavorable side effects, such as tooth and dental work discoloration, dry mouth, discomfort in the mouth, accelerated supragingival calculus growth, and altered taste. Cinnamon extract is one such chemical. It is well recognized to have a number of medicinal properties and could serve as a cost-efficient and clinically effective mouthwash [29,30].

It was found that the difference in values of periodontal index and gingival index at baseline and one month follow up was statistically meaningful in all categories (p <0.05). In each category there was decrease in values of periodontal index and gingival index at one month follow up. When there was comparison among five categories, the difference in values of periodontal index and gingival index was not meaningful at baseline, however the difference in values were statistically significant at one month follow up. The values were maximum in category V (distilled water) and minimum in herbal mouthrinse (cinnamon, curcumin). The values of conventional mouthrinse (chlorhexidine, povidone iodine) were lesser than category V (distilled water) but greater than herbal mouthrinse (cinnamon, curcumin). However, the difference in values in conventional therapeutic agents (chlorhexidine, povidine)and herbal mouthrinse (cinnamon and curcumin was not statistically significant.

The results are similar to that of previous studies [27, 28, 31, 32, 36] where conventional mouthrinse showed lesser efficiency as compared to herbal mouthrinse. The findings of the current investigation, however, are in conflict with those of prior study [33], which indicated no therapeutic advantages on using chlorhexidine as an ultrasonic cooler over water. However, it should be noted that, in contrast to individuals with advanced periodontitis, the patients included in our current study were those who had been diagnosed with gingivitis. The healing response for both groups of patients vary greatly, therefore this may have had an impact on the study's findings.

In this study, there was reduction in CFU in both conventional therapeutic agents (chlorhexidine, povidine) and herbal mouthrinse (cinnamon and curcumin) study participants. The antibacterial effects of both conventional therapeutic agents (chlorhexidine, povidine) and herbal mouthrinse (cinnamon and curcumin) were comparable. It was observed that CFU values were maximum in control (distill water group) at all locations. The findings were meaningful statistically (p<0.05).These findings are consistent with a prior study [31,35, 37] who discovered that, when used as an ultrasonic cooler, chlorhexidine induced the greatest decrease in CFU numbers as compared to DW and povidone-iodine. A previous study showed there was no significant change in the CFU counts between the patients using either chlorhexidine or essential oils [34] were consistent with these findings.

The CFU values were low in category II (cinnamon), category IV (curcumin) at chest area. The CFU values were low in category I (chlorhexidine) at left and right side of chest. The findings were meaningful statistically (p<0.05). The maximum number of CFU was observed at chest area of patient in all five experimental materials. The findings were meaningful statistically (p<0.05). These results are in agreement with a previous study conducted by other researchers [27,28] who also found that the highest CFU counts were seen on the blood agar plates placed at the patient's chest area.

There was reduction in CFU waterline of dental units in both chlorhexidine and cinnamon study participants. The antibacterial effects of both chlorhexidine and cinnamon were comparable. The maximum CFU effect was seen in distilled water category while it was minimum in cinnamon category. The findings were meaningful statistically. The values were greater in chlorhexidine category as compared to cinnamon. However, the difference in values in cinnamon and chlorhexidine did not show statistically significant difference. The results are similar to that of previous studies [27, 28,31]. The results of studies on cinnamon's and curcumin application in medicine reveal that it has antifungal, anti-inflammatory and antibacterial properties [23]. This data supports the use of extract of cinnamon and curcumin as a mouthwash in the management of gingivitis and for promoting gingival health [24].

## Conclusion:

The effect of both conventional (chlorhexidine, povidone iodine) and herbal mouthrinse (curcumin, cinnamon) on gingival health when used as an ultrasonic cooling agent was almost same. Cinnamon and curcumin owing to their minimal adverse effects and low cost is an alternative to chlorhexidine and povidone iodine for reducing bacterial load in dental aerosols produced due to ultrasonic scalers.

## Figures and Tables

**Table 1:** Values of gingival index at baseline and one month follow up in all five categories

**Experimental group**	**Category I**	**Category II**	**Category III**	**Category IV**	**Category V**	**p-value**
Baseline	2.31±0.21	2.23±0.18	2.29±0.21	2.24±0.18	2.32±0.18	0.24
1 month	0.27 ±0.15	0.20±0.04	0.25 ±0.17	0.21 ±0.04	0.58±0.19	<0.05
Intra-group variation. P-value	<0.05	<0.05	<0.05	<0.05	<0.05	

**Table 2:** Values of plaque index at baseline and one month follow up in all five categories

**Experimental group**	**Category I**	**Category II**	**Category III**	**Category IV**	**Category V**	**p-value**
Baseline	2.42±0.23	2.38±0.31	2.42±0.21	2.38±0.34	2.51±0.07	0.11
1 month	1.10±0.10	1.06±0.12	1.12±0.10	1.07±0.12	1.31±0.14	<0.05
Intra-group variation	<0.05	<0.05	<0.05	<0.05	<0.05	

**Table 3:** CFU at agar plates on different locations

**Location of the plate**	**Patient's chest area**	**Patient's right side**	**Patient's left side**	**P value**
Category I	627.36 ±34.11	407.7 ± 25.88	403.56±16.94	<0.05
Category II	576.8 ± 30.42	419.6±48.22	413.6 ±51.38	<0.05
Category III	620.47 ±35.22	411.9 ± 26.99	404.67 ±17.05	<0.05
Category IV	589.94± 31.53	421.7±49.33	416.72±52.49	<0.05
Category V	1396.16 ±214.94	1064.06±26.70	1009.86 ±23.31	<0.05
P	<0.05	<0.05	<0.05	

**Table 4:** CFU in waterlines of dental units

**Experimental category**	**CFU**
Category I	361.81±15.21
Category II	279.81 ±27.81
Category III	354.81±16.32
Category IV	281.92 ±28.92
Category V	680.1 ±42.51
P	<0.05
